# Ionotropic Gelation of Chitosan for Next-Generation Composite Proton Conducting Flat Structures

**DOI:** 10.3390/molecules25071632

**Published:** 2020-04-02

**Authors:** Patrizia Bocchetta

**Affiliations:** Dipartimento di Ingegneria dell’Innovazione, Università del Salento via Monteroni, 73100 Lecce, Italy; patrizia.bocchetta@unisalento.it; Tel.: +39-328-922-0106

**Keywords:** chitosan, composite gel structures, proton conducting materials, ionotropic gelation, fuel cell membranes

## Abstract

(1) Background: Ionotropic gelation of cost-effective and eco-friendly biopolymer chitosan (Chit) is a novel and promising approach to the one-step synthesis of proton-conducting fuel cell bio-membranes.The method discovered by the author in 2011 and subsequently drowned among very few papers. This work aimed to relaunch this method through clear and effective communication of new unpublished results emphasizing the key aspects of this topic for successful dissemination of the results and significant future developments. (2) Methods and results: The mechanism of in-situ ionotropic gelation of Chit on an alumina substrate by phosphotungtate anions (PWA^3−^) was discussed and analyzed. The study sheds light on the effect of prolonged post-treatment in phosphotungstic acid (PWA) solution on the obtained chitosan/phosphotungstate (Chit-PWA) flat structures. Methods used included combined structural (XRD), thermal-gravimetric (DTG), electrochemical (in-situ EIS), compositional (EDX),morphological analysis (SEM), as well as the performances in a low temperature H_2_/O_2_ fuel cell(4) Conclusions: This contribution discloses novel possibilities aimed at increasing the impact of ionotropic gelation of chitosan on the scientific community working on the synthesis of novel proton conductive bio-composite membranes and structures.

## 1. Introduction

### 1.1. Why Chitosan?

**Figure 1:**
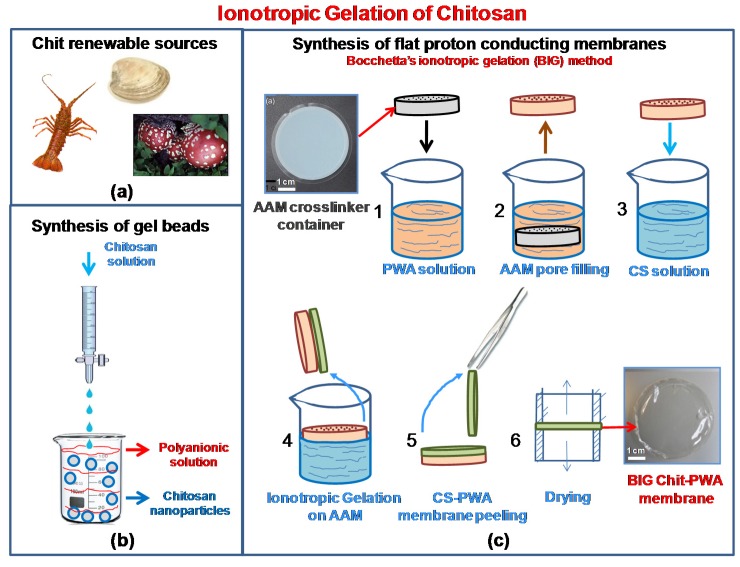
Chitosan renewable sources (**a**); Scheme of ionotropic gelation of chitosan through cross-linking for the formation of (**b**) gel beads; (**c**) proton conducting flat structures (BIG method).

Among various biopolymers compliant with ionic crosslinking, chitosan represents the most promising one [1]. This is due to its several suitable properties. (i) It is a renewable material derived from the alkaline deacetylation of chitin, one of the most abundant natural polysaccharides on Earth [2]. It is found in crustacean shells, insects, molluscan, and fungal mycelia (Figure 1a). (ii) Chit is cost-effective, eco-friendly, bio-compatible, and has a low permeability to methanol [3,4]. (iii) Unlike chitin, Chit is soluble in water under slightly acidic conditions due to the presence of ammine groups in diluted organic acids [5,6,7]. This allows gel formation in various configurations [8]. (iv) It has good film-forming properties due to the presence of intra- and inter-molecular hydrogen bonding [9]. (v) The presence of polar functional groups in the structure, specifically hydroxyl (-OH), primary amine (-NH_2_), and ether (C-O-C) groups, facilitates the chemical modifications of chitosan and the tailored synthesis of Chit-based materials for specific applications [10,11,12]. Owing to the very low proton conductivity of pristine chitosan (about 1.2 × 10^−2^ S cm^−1^ at 25 °C [8]), chemical modification of the polymer has been intensively exploited in recent years to generate ion-exchange sites to improve proton/ionic conductivity of the composite material [13,14,15,16]. 

### 1.2. Why Phosphotungstic Acid?

It is well known that phosphotungstic acid (PWA) has higher proton conductivity than other inorganic solids in its fully hydrated state (**σ** = 1.7 × 10^−1^ S·cm^−1^ at 25 °C [17,18]). It also has non-corrosive and environmental-friendly features. For these reasons, PWA has been intensively studied as a promising material for fuel cell membranes. However, its low mechanical resistance and high solubility in water produced at the cathode by oxygen reduction reaction sharply limits the durability of the fuel cell power output [19,20,21]. To overcome these issues, PWA and heteropolyacids (HPA), need a physical or chemical bonded supporting matrix. This is well detailed in a recent review of the progress of HPA-based composite proton-conducting membranes [22]. Among the different approaches used to reduce HPA dissolution and leakage, the capability of PWA to form an insoluble complex with chitosan through strong electrostatic interactions was proposed in 2007. It involves a two-step procedure consisting of the immersion of the pre-formed Chit membrane in PWA aqueous solution [23,24]. Still, a few papers have developed this promising strategy with novel approaches [21,25,26].

### 1.3. Methods for the Fabrication of Proton Conducting Chitosan-Based Membranes

In the past decade, chitosan-based membranes for fuel cell applications have been developed using different methods [16,27,28,29]. The low proton conductivity of pristine chitosan has been improved to high values by doping the bio-polymer with inorganic fillers (cesium phosphotungstate salt [30], silica-supported silicotungstic acid [31], or calcium oxide [32]). It can also be improvedby chemically modifying chitosan (sulphonation, phosphorylation, quaternization, and cross-linking [17,28,29,31,33]). Flat proton-conducting chitosan membranes have been obtained typically by solution-cast techniques [16,23,24,32,33] and chemical neutralization [34,35]. In contrast, ionotropic gelation is a procedure not deservedly developed in fuel cell literature, and thus, is confined to a very few papers [25,26]. Instead, ionotropic gelation is usually obtained by the polyelectrolyte complexation technique or crosslinking where hydrogels are formed by adding one polyelectrolyte to another polyelectrolyte having an opposite charge [36,37]. Given the ability to produce biocompatible materials, ionotropic gelation has been extensively developed in the biomedical field for the synthesis of gel beads, nanoparticles, micro/nanogels, and fibers [38,39,40,41]. The method is schematized in Figure 1b. By adding dropwise under constant stirring, an anionic polyelectrolyte solution, usually alginate, polyphosphates, and organic sulfates, into an acidic chitosan solution, chitosan undergoes ionic gelation forming spherical particles. Instead of tedious cross-linking emulsion procedures, ionotropic gelation to prepare chitosan proton conducting membranes is a straightforward and mild process. The possible application of ionotropic gelation of chitosan to the synthesis of flat membranes with high proton conductivity was proven for the first time by Bocchetta in 2011 [21] and developed in further studies [26,27]. It is relaunched in this paper with new results and insights (Figure 1c).

Ionotropic gelation of chitosan to synthesize proton-conducting membranes offers the advantage of having chitosan synthesis and proton conductive sites generation in one single step. This is because the chitosan crosslinker is also the proton conductivity donor. Therefore PWA^3−^ anions are injected across the polymer bulk phase simultaneously to the Chit gelation, assuring continuity in the through-plane proton transport. On the contrary, solution-cast pre-formed chitosan cannot retain good conductivity because of the limited surface-to-bulk diffusion of functional molecules through the polymer’s matrix. 

In this paper, the mechanism of ionotropic gelation of Chit-PWA membranes was discussed. The paper explains the step by step formation of proton conducting membranes and combined XRD, EDX, SEM, TGA, FTIR analysis, and H_2_-O_2_ fuel cell performance studies. The obtained results allowed the disclosure of novel insights functional to a future quantum leap of ionotropic gelled chitosan membranes for fuel cell application.

## 2. Results and Discussion

### 2.1. In-Situ Ionotropic Gelation of Chitosan with Phosphotungstate

#### 2.1.1. The BIG Method

**Figure 2:**
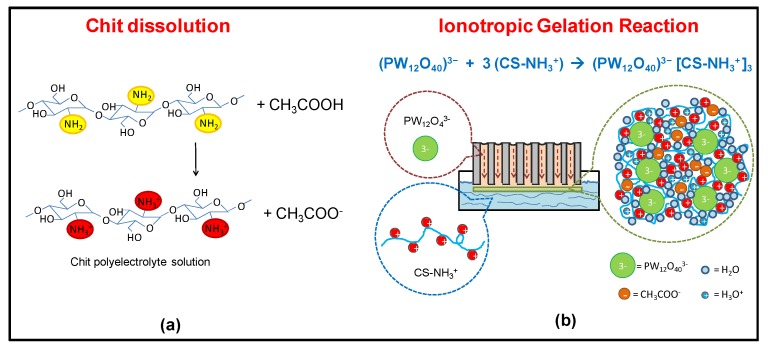
Scheme of the (**a**) chitosan polyelectrolyte formation in acetic acid; and (**b**) in-situ ionotropic gelation of chitosan through cross-linking with PWA^3−^ anions on AAM flat substrate (BIG method, step 4 of Figure 1c).

The Chit-PWA polymeric films were prepared following the experimental conditions of the first synthesis performed by the author in 2011 [21,26,27] (illustrated in Figure 1b and detailed in Section 3.1). Cationic polyelectrolyte chitosan was obtained by dissolving the polymer in slightly acid pH (acetic acid) aqueous solution according to the reaction illustrated in Figure 2a. At neutral pH, Chit existed in the insoluble form Chit-NH_2_ and changed to the protonated soluble CS-NH_3_^+^ form at values ranging from 6.2 to 7. This was due to the protonation of the glucosamine units and the weakling of hydrogen bonding. 

To promote the formation of flat thin-film proton-conducting structures, we used porous anodic alumina. This was because of (i) the high porosity and uniform thickness that make AAM an optimum “container” [42,43] of the ionic cross-linker solution. The solution was allowed to flow from the bottom of the membrane to the top (Figure 2b). (ii) The PWA^3−^ solution coming from the internal porosity distributes over the AAM surface exposed to the Chit solution, allowing the ionotropic gelation reaction to produce flat regular Chit-PWA membranes. This was achieved by simple immersion in the chitosan solution (step 4 of Figure 1c).

Figure 2b shows that negatively charged phosphotungstate polyoxyanions (PW_12_O_40_)^3−^ are amenable to ionically crosslink the positively charged –NH_3_^+^ groups of Chit at the AAM/Chit solution interface. Thus, forming a flat three-dimensional lattice of proton-conducting composite film adherent to the AAM substrate. The formation of ionic bonds, supported by hydrogen bonds between the O-H groups of CS, created a reticular network of an immobilized polymer leading to a gelled polymeric film that could be peeled off from the AAM support (steps 5–7 of Figure 1c). 

#### 2.1.2. Monitoring the BIG Reaction and the Prolonged Post-Crosslinking Treatments

**Figure 3:**
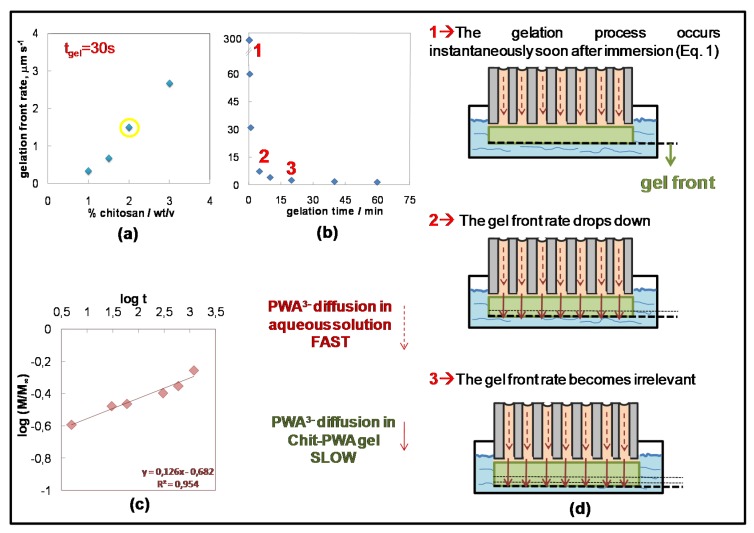
Gelation front rate as a function of (**a**) chitosan concentration; and (**b**) gelation time at 2% *wt*/*v* Chit. (**c**) Curves of $\log\left(\frac{M_{t}}{M_{\infty }}\right)$ versus log(t) for Chit-PWA composite structures at 2% *wt*/*v* Chit. The linear equation can be applied only for log(Mt/M∞) ≤ 0.22. (**d**) Scheme illustrating the growth steps of Chit/PWA composite flat structures over the AAM crosslinker-container (step 4 in Figure 1c)

Figure 3 shows a sketch of the ionotropic gelation evolution with time. The gelation front rate was measured by SEM thickness data of the Chit-PWA membranes formed at different gelation times. It was plotted against (i) the Chit concentration at fixed gelation time (30 s) (Figure 3a) and (ii) the gelation time for fixed Chit concentration (2% *wt*/*v*) (Figure 3b). Three main steps were involved in the ionotropic gelation process. In the first step (named 1 in Figure 3d), i.e., in the first instances of AAM immersion in the Chit solution, PWA^3−^ and Chit-NH_3_^+^ came into contact at the AAM/Chit sol interface and immediately reacted as expected for the electrostatic ionic bonds’ formation. This translated into a high initial gelation front rate (Figure 3b), after which PWA anions diffused into the initially formed polymeric gel and reached the Chit solution, where a new three-dimensional lattice of ionically cross-linked Chit was formed. The gelation front rate drastically decreased, indicating that a new process controlled the gelation kinetic (the second step, named 2 in Figure 3d). Finally, at about 20 min of ionotropic gelation, the gelation front rate became insignificant (the third step, named 3 in Figure 3d). 

To show the kinetically controlling phenomenon, we analyzed the different processes involved in the ionotropic gelation of the Chit. These included (a) the chemical reaction between PWA^3−^ and the Chit polymer chains. (b) The transport of PWA^3−^ anion from the bulk of the phosphotungstic acid solution towards the CS/PTA interface. (c) The transport of PWA^3−^ anions inside the already gelled film. The electrostatic chemical reaction between PWA and Chit and the diffusion of PWA^3−^ anion from the bulk of the PWA solution inside the AAM pores toward the Chit/PWA interface in highly concentrated (0.76 M) PWA solutions were expected to be very quick, and thus, not controlling. Therefore, it may be hypothesized that the gel formation rate was controlled by the diffusion rate of the PWA^3−^ anion through the already gelled CS/PTA layer formed in the first seconds at the contact interface (from top to bottom).

This hypothesis is confirmed by the experiments carried out at a constant concentration of PWA (0.76 M) and variable concentration of the Chit solution in the range of 1–3 *wt*/*vol*%. The stoichiometric quantity of PWA necessary to crosslink all the CS-NH_3_^+^ groups of Chit with a de-acetylation grade of 0.85% (as that employed in this work) was calculated to be 0.034 mol/L. This value was sharply lower than the concentration of 0.76 M used in this work, indicating that Chit represents the limiting reagent of the ionotropic gelation reaction (Figure 2b). Figure 3a shows the thickness of the final CS/PTA films (estimated by direct SEM observation of their cross-section) increasing with an increasing concentration of CS solution. This effect produced a linear trend of the gelation front rate against Chit concentration that agreed with data reported in Reference [26]. By decreasing the concentration of the PWA solution near the stoichiometric value (~0.05 M PWA for Chit 2% *wt*/*v*), the mechanical properties of the final gels were not sufficient to self-sustain the membrane and to allow the peeling of the film from the AAM support. 

Two considerations arise from these results, i.e., (i) not all the PWA^3−^ anions were prone to crosslink chitosan despite a highly concentrated PWA solution. (ii) The crosslinking of –NH^3+^ sites was essential to stabilize the three-dimensional structure of the chitosan chains providing mechanical resistance [44]. Immersions in highly concentrated PWA solution after the BIG synthesis have been applied for prolonged times unlike previous data [25,26]. They were used to investigate the effect of crosslinking grade on the BIG Chit/PWA membranes features.

Stated that the transport of PWA^3−^ anions inside the already gelled film may be considered as the process controlling the Chit gelation rate, an opening discussion on the transport mechanism during the BIG process (Figure 3d) is presented according to the experimental data. In a first approach, we can assume that the PWA^3−^ concentration distribution is homogeneous through the chitosan membrane, the solvent absorption and solute diffusion are simultaneous processes and the diffusivity is unidirectional and perpendicular to the surface of the Chit-PWA thin film (plausible for our high aspect ratio samples [45]). In these conditions, the PWA^3−^ diffusion inside the Chit-PWA hydrogel could be approximately determined by a simple semi-empirical equation derived from the one-dimensional Fick’s second law of diffusion and commonly used in literature to describe the solute release in hydrogels [46,47], specifically in chitosan [48]:

(1) $$\frac{M_{t}}{M_{\infty }}={\mathrm{kt}}^{n}$$

where Mt and M∞ represent the amount of PWA solution diffused into the gel at time t and at an infinite time (equilibrium state), respectively. k is a constant related to the structure of the network, and n is the diffusion exponent that gives information on the mechanism of release. The number n was estimated by fitting the experimental data plotted as $\log\left(\frac{M_{t}}{M_{\infty }}\right)$ versus log(t) (Figure 3c) to obtain information on the mechanism of PWA^3−^ solution diffusion. According to the recorded data shown in Figure 3c, the estimated exponent n value was 0.13, which was below 0.5 marking a pure Fickian diffusion. This condition, called the ‘Less Fickian’ behavior, is reported to occur when the penetration rate of the considered species (PWA solution in this work) is much below the polymer chain relaxation rate [49,50]. It must be considered that two types of diffusion generally govern the rate and amount of PWA solution penetration into a gel of planar geometry. One controlled by the concentration gradient and the other by the polymer relaxation phenomena [50,51,52,53]. The ‘Less Fickian’ behavior of the Chit-PWA membranes corroborated with the gelation front rate abrupt decrease recorded as a function of the gelation time and reported in Figure 3b. It is likely that the initially instantaneous Chit-PWA polymer film drastically slowed down the membrane growth due to the chitosan chain relaxation processes. The PWA^3−^ transport inside the film was delayed significantly, as well as the ionotropic reaction occurring at the Chit-PWA film/Chit solution interface, where active fresh –NH_3_^+^ sites were available (see the sketch of Figure 3). 

**Figure 4:**
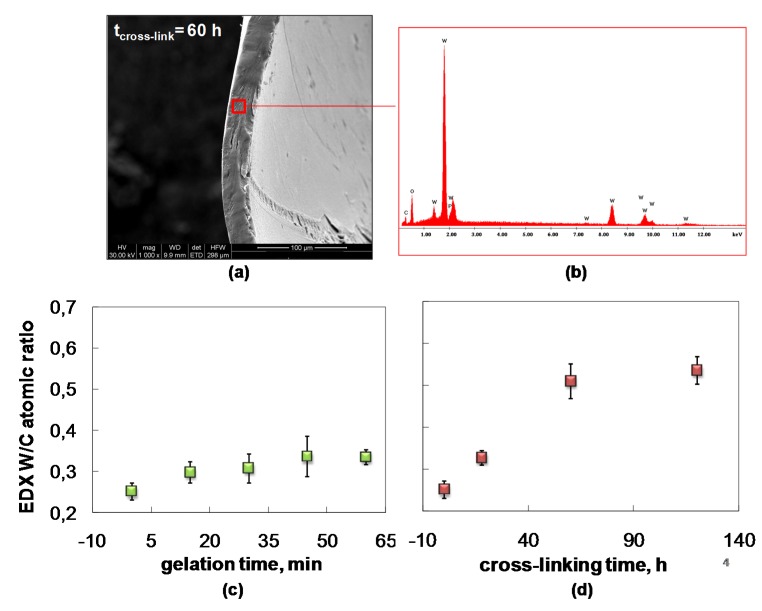
Composite Chit/PWA flat structures prepared according to theBIG method at 2% *wt*/*v* Chit. (**a**) Typical SEM cross-section; (**b**) typical EDX spectrum; EDX quantitative W/C atomic ratio versus (**c**) BIG gelation time and (**d**) post crosslinking time.

This situation agreed with the EDX analysis carried out in different areas of the sample cross-sections (Figure 4a,b). 

Information on the PWA^3−^ contents of the Chit membrane during the ionotropic gelation process and the post-crosslinking step could be extracted using EDX quantitative estimation of the W/C atomic ratio (Figure 4c,d). Figure 4c shows that the *W*/*C* atomic ratio moderately increases in the first minutes of Chit-PWA gelation. After 30 min, it remains quite constant and in good agreement with the diffusion mechanism of the PWA solution inside the Chit-PWA film discussed above. The crosslinking reaction likely requires low gelation rates to be more incisive. From the EDX W/C atomic ratio data of Figure 4d, we detected that the crosslinking grade notably increased going from as-formed Chit-PWA to 60 h post-crosslinked Chit/PWA film. Afterward, it remained quite constant, suggesting that complete ionic cross-linking of the –NH_3_^+^ sites occurred at 60 h of immersion. 

**Figure 5:**
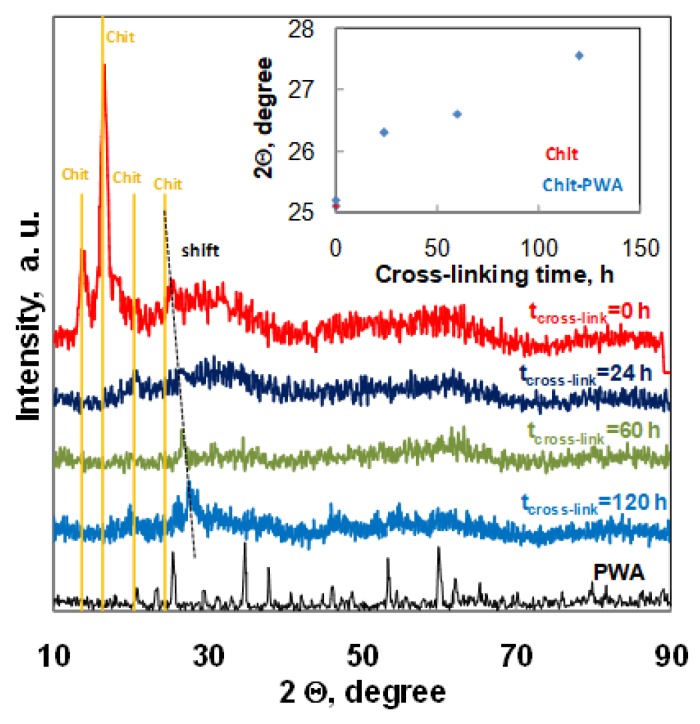
XRD spectra recorded for composite Chit/PWA flat structures prepared according to the BIG method at 1% *wt*/*v* Chit and different post crosslinking times.

These data are also in agreement with the XRD analysis of Chit-PWA membranes treated at different post-crosslinking immersion times up to 120 h (Figure 5). 

The diffractograms of Chit-PWA membranes soon after the ionotropic gelation step (t_cross-link_=0) exhibited four major crystalline peaks at 2Ө values of about 13.7°, 16.4°, 20.5°, and 25.4°. According to literature data, the peaks at 13.7° could be attributed to annealed polymorph chitosan [54], with the peak at 16.4° to Form I polymorph chitosan [55] and the peaks at 20.5° and 25.4° to Form II polymorph chitosan [56,57]. The partial crystalline nature of chit was related to the inter-and intra-molecular hydrogen bonding between the amino groups and hydroxyl groups. After post-crosslinking immersion in the PWA solution, the peaks at 13.7° and 16.4° disappeared. This effect showedthat the strong interactions between chitosan NH^3+^ groups and (PWA)^3−^ anions considerably modified the functional groups of the polymer by deprotonation of the amine groups and loss of hydrogen bonding. This modification reduced the crystallinity of chitosan and agreed with the recognized dependence of the ionic conduction of chitosan electrolytes of the amorphous rather than crystalline phase [58], The peak at 2Ө = 20.5 remained constant in intensity and position, while the peak recorded at 25.4 for pure Chit film gradually shifted toward higher 2Ө values as the crosslinking immersion time increased (inset of Figure 5).This result indicated a trend in the polymer arrangements related to the formation of Form II polymorph chitosan in agreement with previous papers [26]. It is worth noting that the peak at 25.4 °C (characteristic of Chit treated at t_cross-link_ = 0) reappeared at t_cross-link_ = 120 h next to the shifted-one at 27.7 °C. This suggested that the chitosan crosslinking treatment prolonged at 120 h induced additional re-arrangements to the structure of the polymer, in agreement with the correspondent decrease of crosslinking grade and proton conductivity documented by the EDX, EIS, and Fuel Cell performance results. 

The absence of PWA peaks in the Chit-PWA polyelectrolyte film confirm that PWA reacted with chitosan forming an insoluble complex [23,24].

**Figure 6:**
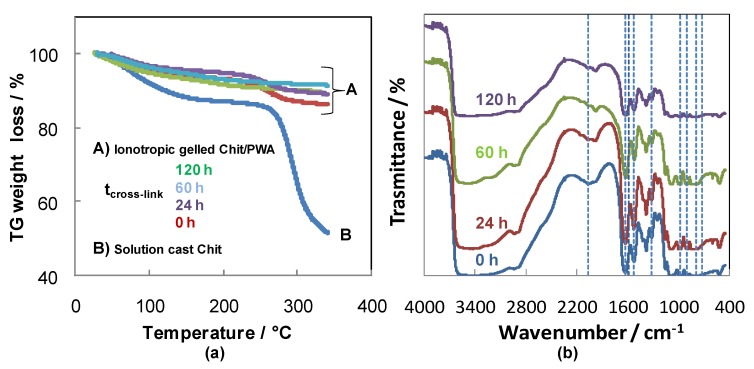
(**a**) TG under N_2_ atmosphere and (**b**) FTIR analysis of composite Chit/PWA flat structures prepared according to the BIG method at 1% *wt*/*v* Chit and different post crosslinking times.

These results corroborate further with thermo-gravimetric analysis of the Chit-PWA film performed with a 10 °C/min heating rate under nitrogen (Figure 6a). 

The weight loss below and around 100 °C for the samples can be attributed to water evaporation. By comparing pure Chit and BIG cross-linked chitosan, a notable improvement in thermal stability appeared from the weight loss in the range of 250–350 °C. Two stages of thermal degradation can be detected for solution-cast Chit, pristine and post-crosslinked for 24 h BIG Chit-PWA composites. The first stage took place between 40 and 130◦C with an almost 15% loss of the initial weight, attributed to evaporation of the solvent traces (acetic acid and water), used for chitosan film preparation. The second decomposition stage of the membranes started above 180 °C and extended to about 300 °C. The most important mass loss (45%) was recorded for solution-cast chitosan. It is attributed to dehydration of the saccharide rings, depolymerization, and decomposition of the acetylated and deacetylated units of the polymer [59]. By prolonging the post crosslinking treatments (60, 120 h) of the BIG Chit-PWA samples, the lowest weight loss could be interpreted as further evidence of an increase of crosslinking grade between the Chit chains and PWA^3−^ polyanions. The use of PWA cross-linker was considered more promising concerning sulphuric acid, which produced cross-linked Chit with thermal behavior similar to unbonded Chit [60].

The post-crosslinking reaction was followed by FTIR analysis of the Chit-PWA structures, as depicted in Figure 6b. According to the assignments reported in Reference [61], the absorption bands at 1081, 985, 892, 792 cm^−1^,recorded in all the spectra, were attributed to the ν (P-O), ν (W-Ot) (Ot = terminal oxygen), ν (W-Oe-W) (Oe = edge oxygen), and ν (W-Oc-W) (Oc = corner oxygen), respectively. This result suggests that the geometry of the Keggin ion was maintained after the ionotropic gelation of chitosan and the subsequent crosslinking reaction. Evidence of the columbic interaction between amino groups of Chit and PWA was found. It included (i) the blue shift of the peaks assigned to both the bending mode of the W–Ob–W bridging bond and stretching vibration of the W-Ot terminal bond (from 1310 to 1320 cm^−1^). (ii) The presence of the absorption bands characteristic of NH_3_^+^ bending vibrations at 1630 cm^−1^ and 1528 cm^−1^ instead of values relating to NH_2_ (1587 cm^−1^). (iii) The comparison between IR spectra of pristine chitosan [62] and composite Chit/PWA membranes. It can be observed that in the 3500–2500 cm^−1^ region, the chitosan O-H (3400 cm^−1^) and N-H (3200 cm^−1^) absorption bands gradually became less distinct and broad with increases in the post-crosslinking step time up to 120 h. This band broadened by increasing the post-crosslinking time, and it was related to the rise in the cross-linking grade because of the strong interaction between the (PWA)^3−^ and NH_3_^+^ ions. As the reaction time increased, more ions interacted with the -NH_3_^+^ groups, so the band at 3200 cm^−1^ continued to broaden. In addition, the position of the absorption due to the C-H stretching vibrations shifted to higher wavenumbers (from 2916 and 2867 cm^−1^ (pristine chitosan [62]) to 2934 and 2890 cm^−1^, respectively. All these results indicated the presence of interactions between phosphotungstic acid and the NH_3_^+^ groups of chitosan and corroborated with the FTIR data of Chit crosslinked by H_2_SO_4_ [63].

**Figure 7:**
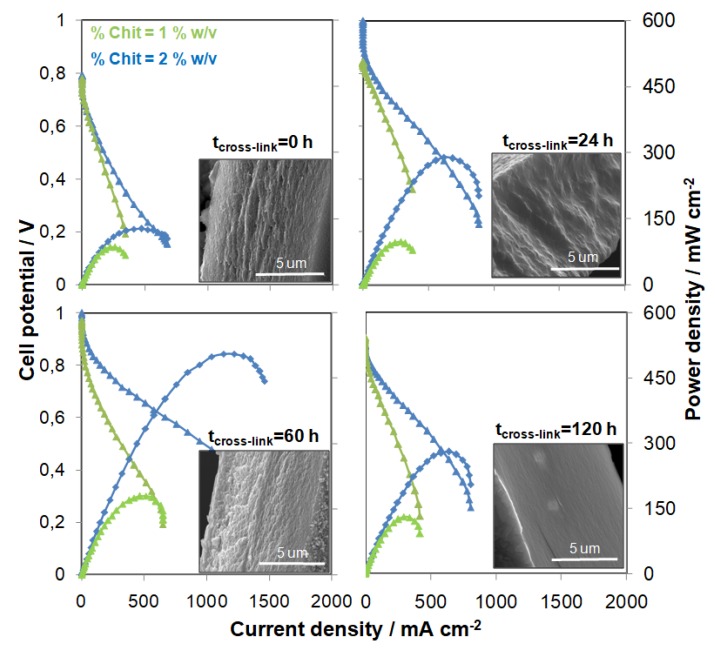
Polarization and power curves recorded for an H_2_/O_2_ fuel cell working at 25 °C cell and gas temperature and 1 mg cm^−2^ Pt loading with composite Chit/PWA flat membranes prepared according to the BIG method at 1 (green line) and 2 (blue line)%*wt*/*v* Chit and different post-crosslinking step times (0, 24, 60, 120 h). Insets: Correspondent Chit/PWA flat membrane SEM cross-sectional views.

In Figure 7, the polarization curves and power output relating to two different Chit-PWA membranes (BIG in 1 and 2 *wt*/*v*% Chit) are reported for four different post-crosslinking times (panel (a) 0 h; panel(b) 24 h; panel(c) 60 h; and panel (d) 120 h). The power peak increased as the crosslinking time increased up to 60 h, and afterward, it slightly decreased for both the Chit-PWA membrane samples. These data were consistent with the EDX data showing that 60 h are required to complete the crosslinking process of Chit chains with PWA^3−^ anions from the ionotropic gelation step (Figure 1b). As evidenced in all the recorded polarization curves, kinetic was governed mainly by the ohmic overpotential (the linear part of the curve) attributed mostly to the membrane proton conductivity.

SEM cross-sections of as-gelled and post-crosslinked Chit/PWA flat structures are reported in the insets of Figure 7. The images show a regular surface without significant cracks or holes typically observed in chitosan film prepared through solution-cast or other coagulation methods [64]. This result indicated that PWA^3−^ crosslinker exhibited good intermolecular interactions and compatibility with chitosan polymer. The ionotropic gelation method was capable of producing a uniform distribution of crosslinks as expected for a single step crosslinking-synthesis process.

A detailed inspection of the internal morphologies also revealed a lamellar structure in agreement with typical crosslinked chitosan structures [65], and textured and rough features disappeared with increases in the cross-linking grade. The absence of PWA precipitates confirmed that the acid gave way to electrostatic interactions with chitosan agreeing with the XRD, FTIR, and TG results discussed above. It is worth noting that the post-crosslinking process did not affect the thickness of the Chit-PWA membranes.

**Figure 8:**
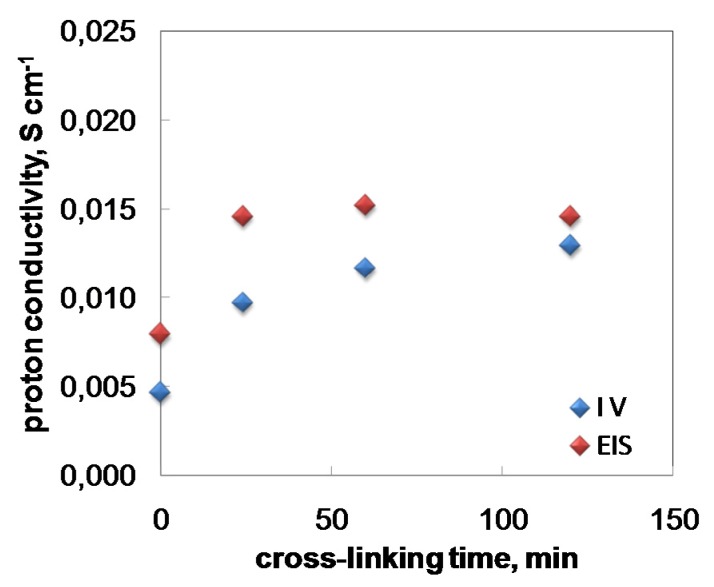
Proton conductivity values versus post-crosslinking times of composite Chit/PWA flat membranes prepared according to the BIG method at 2% *wt*/*v* Chit.

Estimation of Chit/PWA membrane proton conductivity was obtained through in–situ ACimpedance measurements carried out in a single fuel cell fed with H_2_/O_2_ at 25 °C gas and cell temperature and open circuit potential conditions (Figure 8). We observed that the σ_m_ values obtained by EIS were consistent with the values obtained by analyzing the fuel cell polarization curves in the pseudo-linear part of the curve (IV) [25].

The EIS proton conductivity increased as the post-crosslinking treatment time increased to 60 h and then slightly decreased at 120 h. This result confirmed that the crosslinking grade of Chit-PWA membranes and the fuel cell peak power were strictly correlated.

## 3. Materials and Methods

### 3.1. Materials

Commercial anodic alumina membranes (AAM) Anodisc-47 with pore diameters 200 nm, porosity 43%, and thickness 50 μm (Whatman® Anodisc inorganic filter membrane), chitosan powder with an de-acetylation grade of 0.85%, acetic acid, and phosphotungstic acid were provided by Sigma-Aldrich, division of Merck KGaA, Darmstadt, Germany. E-Tek Inc. (Natick, MA) provided carbon paper electrodes Toray 40% wet Proofed. The catalytic layer was prepared by spreading a mixture of Pt black/C black (30 % Pt on Vulcan XC-72, E-Tek) stirred in n-butyl acetate. This was done for at least 3 h on the electrodes up to a 1 mg cm^−2^ loading of black Pt. AirLiquide (Milano, Italy) provided oxygen (99.5 % purity, 1 bar) and hydrogen (99.5 % purity, 1 bar).

### 3.2. The Bocchetta’s Ionotropic Gelation (BIG) Procedure

Figure 1b shows the experimental procedure followed to synthesize flat proton-conducting Chit/PWA structures by ionotropic gelation (BIG process). Commercial AAM was used as a porous matrix containing the crosslinker solution. Chit was solubilized in distilled water and acetic acid (1% *v*/*v*) to achieve concentrations of 1, 2, and 3% *wt*/*v*. The solution is stirred for at least 24 h before use and then filtered to remove impurities. Highly concentrated PWA aqueous solution (0.76 M), containing only (PW_12_O_40_)^3−^ anions [66] was used as a crosslinker solution.

AAM matrix (step 1, Figure 1c) was immersed in the PWA aqueous solution to embed the porous structure (step 2, Figure 1c). Afterward, the AAM-PWA sample (step 3, Figure 1c) was dipped in the Chit solution through only one face, as shown in step 4 of Figure 1c. During this step, Chit cationic polyelectrolyte and the phosphotungstate anions gave place to ionotropic gelation. This formed a proton-conducting self-standing Chit-PWA membrane at the AAM side exposed to the solution (step 5 of Figure 1c). The formation of a flat structure was possible because the chitosan gel was entrained by the viscous drags acting at the AAM substrate. Finally, the obtained self-standing Chit-PWA samples were located between two cylinders to avoid wrinkling phenomena during the drying process (step 6, Figure 1c). After the BIG process, the as-gelled Chit-PWA membranes were chemically treated by immersion in 0.76 M PWA solution for different time intervals (named “post-crosslinking step” in the text) to improve the crosslinking grade of the chitosan.

### 3.3. Characterization of the Chit/PWA Composite Membranes

X-ray Diffraction (XRD) analysis was conducted on a Philips X-Ray Generator (Model PW 1130) with a PW (Model 1050) goniometry (Eindhoven, The Netherlands). Scanning Electron Microscopy (SEM) analysis was performed using a Philips XL30 ESEM coupled with EDX equipment (Amsterdam, The Netherlands). The morphology and compositional features of the samples were analyzed through a Scanning Electron Microscope Philips XL30 ESEM coupled with EDX equipment. Fourier-transform infrared (FTIR) analysis was carried out using a PerkinElmer Spectrum Two spectrometer (Waltham, MA, USA) in the ATR mode in the range 400–4000 cm^−1^. Thermogravimetric (TG) analysis was done using a Netzsch STA/409/2 (Selb, Germany) thermal analysis system at a scanning rate of 10 °C min^−1^ in the range 25–350 °C under a nitrogen atmosphere.

### 3.4. Fuel Cell Assembling and Measurements

The composite Chit/PWA membranes synthesized by ionotropic gelation were assembled between two catalyzed carbon paper electrodes. The Membrane Electrode Assembly (MEA) was assembled between two graphite plates machined with single-serpentine flow channels in a 5 cm^2^ commercial single fuel cell test system (FuelCellTechnologies, Inc., Albuquerque, New Mexico). The fuel cell was fed with oxygen and hydrogen humidified at room temperature. The performance of the fuel cell was evaluated by recording the cell potential as a function of the cell current throw an h-tec Fuel Cell Monitor (item 1950) interface supplied by The Fuel Cell Store, College Station, Texas. 

The through-plane proton conductivity (σ) of the Chit-PWA membranes was tested using the two electrode Electrochemical Impedance Spectroscopy technique using a Parstat 2263 potentiostat equipped equipped with an Impedance Analyzer, Oak Ridge, TN, United States). This was performed under H_2_/O_2_ fuel cell operation in the range of 100 kHz–0.1 Hz at 25 °C, an open circuit potential, and an AC amplitude of 10 mV. Before each measurement, the fuel cell was stabilized for at least 15 min. Data analysis and equivalent circuit fitting were performed using the Power Suite and ZSimpleWin software. The membrane resistance R_m_ was estimated from the high-frequency intercept in the Cole–Cole plot and the proton conductivity (σ, S cm^−1^) calculated using the equation:

(2) $$\sigma =\frac{l}{A\cdot R_{m}},$$

where l (cm), A (cm^2^), and R_m_ (Ω) are the thickness of the membrane (obtained by SEM), the actual cross-sectional area of the membrane, and the membrane resistance (obtained by EIS), respectively.

## 4. Conclusions

In this paper, ionotropic gelation of chitosan for the synthesis of flat proton-conducting structures was relaunched. This relaunch was through a detailed analysis of new unpublished results on the mechanism of chitosan ionotropic gelation on alumina substrate by phosphotungtate anions (the BIG method) and multi-technique composite Chit-PWA characterization (EDX, XRD, TG, FTIR, in-situ EIS, SEM).

The study of the mechanism of the BIG synthesis revealed unknown aspects of the kinetic of Chit-PWA gel growth under selected experimental conditions aimed to obtain reproducible, mechanically stable, and highly proton-conductive polymer gel membranes. The diffusion of PWA^3−^ kinetically controls the BIG synthesis- polyanions through the initial instantaneously-formed chitosan film, and this diffusion process regulates, in turn, the final crosslinking grade of chitosan. These results encourage the use of high PWA concentrations of the starting solution and/or the reduction of the gelation rates during the BIG process.

The prolonged post-crosslinking treatments of the BIG Chit-PWA membranes in high concentrated PWA solution successfully increment the PWA content of chitosan. The result shows that the immersion time and PWA crosslinking grade are strictly correlated to the compositional, structural, thermo-gravimetric, electrochemical, and morphological properties of the membranes. All the characterization techniques used in this paper corroborated to define the optimal post-crosslinking time at 60 h. At this value, the Chit/PWA membranes show the highest PWA content, proton conductivity (1,5 × 10^−2^ S/cm), and power output in a 25 °C H_2_/O_2_ fuel cell (505 mW cm^−2^). These results suggest that future actions taken in the direction of increasing the Chit/PWA crosslinking grade will improve fuel cell power output.

This paper demonstrated that the BIG process is a successful approach to the synthesis of flat proton-conducting membranes. It indicated novel strategies for improvement of the method aimed at increasing the impact of ionotropic gelation of chitosan on the scientific community working on the theme.
